# Associations of different types of physical activity (PA) with musculoskeletal disorders (MSDs) at nine body sites in firefighters

**DOI:** 10.3389/fpubh.2026.1804818

**Published:** 2026-03-30

**Authors:** Wenli Gan, Shaofan Weng, Wei Zhou, Naixing Zhang, Huan Guo, Dafeng Lin

**Affiliations:** 1 Department of Epidemiology and Biostatistics, School of Public Health, Jilin University, Changchun, Jilin, China; 2 Laboratory of Occupational Health Epidemiology, Shenzhen Prevention and Treatment Center for Occupational Diseases, Shenzhen, Guangdong, China

**Keywords:** firefighters, musculoskeletal disorders (MSDs), occupational health, occupational physical activity, physical activity (PA)

## Abstract

**Background:**

Musculoskeletal disorders (MSDs) are one of the global health and economic burdens. They have a high prevalence in occupational populations such as firefighters. Besides poor postures and sedentary work, inappropriate physical activity (PA) is recognized as a relevant risk factor for MSDs. However, the independent associations between different types of PA and MSDs have not been fully investigated.

**Objective:**

We systematically investigated the associations of occupational physical activity (OPA), leisure-time physical activity (LTPA) and transport-related physical activity (TPA) with the onset, pain intensity and frequency of MSDs at nine body sites. We quantified their differential effects, so as to provide scientific evidence for the precise prevention and control of MSDs in high-workload occupational populations.

**Methods:**

Multiple linear regression, logistic regression and polynomial regression were applied to analyze the distinct effects of different types of PA (OPA, LTPA, TPA) on the onset, intensity, frequency and cumulative number of painful sites of MSDs at nine body sites.

**Results:**

OPA was associated with the risks of the onset, increased pain intensity and prolonged duration of MSDs at nine body sites (*p* < 0.05). Only high-level LTPA was associated with the onset and increased pain intensity of MSDs at specific body sites (*p* < 0.05). In the state of high-intensity OPA, it was associated with the exacerbated accumulation of MSDs at multiple body sites.

**Conclusion:**

High OPA was a key risk factor for MSDs, and high LTPA was a risk factor for MSDs at specific body sites.

## Highlights

- High-intensity occupational physical activity (OPA) is the strongest risk factor for the prevalence, pain intensity, and pain frequency of musculoskeletal disorders (MSDs) at nine body sites in high-workload firefighters.
- High-intensity LTPA is a risk factor associated with the onset and pain intensity of MSDs at specific body sites, while transport-related physical activity (TPA) has no significant independent association with MSDs.
- As the intensity of OPA increases, the risk of accumulating more painful sites becomes stronger.

## 1 Introduction

Musculoskeletal disorders (MSDs) represent a major global public health challenge. Characterized by pain and functional impairment, MSDs affect multiple body sites including the neck, shoulders and lower back, and are the leading cause of non-fatal disabilities worldwide. By 2021, MSDs had caused approximately 11,850 deaths and 161.9 million disability-adjusted life years (DALYs) globally, imposing a heavy burden on healthcare systems and socioeconomic development (1–4). The prevalence of MSDs in occupational populations is significantly higher than that in other groups, with over 50% of workers worldwide affected (5). Such work-related musculoskeletal disorders (WMSDs) are the primary occupational health issue and a leading cause of disability across the globe (5–8). The age-standardized prevalence rate of MSDs in first-tier cities of China has reached 36.7% (9). Firefighters, as a population engaged in high-intensity physical work involving heavy lifting and repetitive physical exertion, are a typical high-risk group for MSDs. Domestic studies have shown that the prevalence of low back pain among this population is 47.1%, knee pain 40.6, and 50.3% of participants suffer from pain in three or more body sites (10).

Physical activity (PA) is defined as any bodily movement produced by skeletal muscle contraction that requires energy expenditure (11). Appropriate PA can reduce the risk of various diseases, such as cancer, diabetes and depression (12–20). Inappropriate PA, however, increases the risk of MSDs via pathways including muscular overloading and inflammatory responses (21–25). Before 2010, the World Health Organization (WHO) did not classify PA by specific domains. In recent studies, the correlation between different types of PA and health outcomes has been proven to vary across domains, yet the specific association with MSDs remains unclear. Limited existing evidence suggests that excessive physical exertion (e.g., lifting, pushing and pulling heavy objects) and repetitive movements at work may be associated with the onset of WMSDs. A classic quantitative study showed that the prevalence odds ratio of WMSDs for tasks with high repetition and high force (e.g., repetitive heavy lifting) was as high as 30.3, significantly higher than that for other task types (26). Surveillance data from the US mining industry also confirmed that material handling, the most common type of occupational physical activity (OPA), accounts for 23% of all causative factors for WMSDs, primarily affecting the lower back (47%), knees (36%) and shoulders (17%) (27). In other words, OPA is associated with the onset of MSDs, especially WMSDs. However, the dose–response relationship between OPA and MSDs has not been reported to date, and further analysis of the pain intensity and frequency of MSDs is lacking. Research on the associations of leisure-time physical activity (LTPA) and transport-related physical activity (TPA) with MSDs is scarce and controversial both domestically and internationally. Some studies have indicated that LTPA of a certain dose (e.g., ≥3 h of moderate-intensity exercise per week) can alleviate the pain intensity of neck and back MSDs by improving physical and mental health. Particularly in sedentary occupational populations, high LTPA (≥10 h of physical activity per week) can reduce the risk of sedentary-related neck and shoulder pain by 30%, while this protective effect is significantly attenuated or even abrogated in populations with high OPA (28). TPA may exert a weak protective effect on the onset of MSDs, but this association has no statistical significance (29).

Current research still has several gaps. Firstly, most studies analyze PA as an overall exposure variable, ignoring the differential effects of OPA and LTPA on MSDs due to the lack of independent domain distinction between OPA, LTPA, and TPA. This limitation obscures the true effects of PA in different domains, creating a theoretical gap in domain-specific PA analysis, which prevents the accurate identification of distinct health risks. Secondly, among the few studies mentioning domain differences in LTPA, the true risk effect of high-intensity LTPA is not distinguished; in addition, research on TPA is almost non-existent, and the existing conclusions cannot support the precise prevention and control of MSDs in high-load populations. Thirdly, the coverage of nine common MSDs-affected body sites is incomplete, and a lack of targeted studies on high-workload occupational populations. To address these research gaps, the present study took firefighters as the research population, systematically investigated the associations of OPA, LTPA and TPA with the onset, pain intensity and frequency of MSDs at nine body sites, and quantified their differential effects. This study aims to provide scientific evidence for the precise prevention and control of MSDs in high-workload occupational populations.

## 2 Methods

### 2.1 Data sources and samples

A cross-sectional survey was conducted in Shenzhen in October 2024, with the study population comprising on-the-job frontline male firefighters across all administrative districts of the city. A two-stage cluster sampling method was employed: the first stage designated nine administrative districts of Shenzhen (Futian District, Luohu District, Nanshan District, Yantian District, Baoan District, Longgang District, Longhua District, Pingshan District, and Guangming District) as primary sampling units (PSUs), and all districts were fully included to ensure sample coverage across different regions of the city; the second stage regarded all fire brigades, squadrons, and stations within each included administrative district as secondary sampling units (SSUs), and random sampling was used to select some fire stations from each district as survey sites, with a questionnaire survey administered to all study subjects meeting the inclusion criteria in each selected fire station. The sample size was calculated using the formula: $n=\text{DEFF}\times \frac{\left(Z_{a/2}^{2}\times P(1-P)\right)}{\delta^{2}}$ , *Z*^2^_*α*/2_ = 1.96 (two-tailed test), *P* is the expected prevalence of MSDs (set at 0.45 with reference to previous literature), *δ* = 0.05, and DEFF = 2.0 was adopted as a conservative estimate due to cluster sampling; considering a 10% non-response rate and data missing, a minimum of 845 samples was required after final calculation. A total of 5,690 questionnaires were actually completed (all completers signed written informed consent forms); samples with missing data on sociodemographic characteristics, health characteristics, MSDs, and PA-related variables were excluded, and finally 5,522 eligible subjects were included in the study. The inclusion criteria for this study were: ① on-the-job frontline male firefighters in Shenzhen; ② aged ≥ 18 years; ③ normal cognitive function. The exclusion criterion was: any missing data on sociodemographic characteristics (such as age and ethnicity), health characteristics (such as BMI and smoking/drinking history), MSDs pain indicators, and PA-related data in the questionnaire.

### 2.2 Exposure

Exposure variables included three types of physical activity: OPA, LTPA, and TPA. The Chinese validated version of the International Physical Activity Questionnaire-Short Form (IPAQ-SF) was used to assess physical activity, and the energy expenditure of PA in the three domains (OPA, LTPA, and TPA) was further evaluated based on metabolic equivalents (METs). One MET is defined as the energy expenditure at resting sitting posture (1 kcal/kg/h). Moderate-intensity activities were assigned a value of 4 MET, and vigorous-intensity activities a value of 8 MET. Only activities lasting for at least 10 min in a single session were included. When participants reported an activity duration of more than 180 min per day, the duration was truncated at 180 min. Energy expenditure in each domain was standardized as follows: OPA and LTPA: total energy expenditure was expressed as MET-min/week, calculation formula: Total MET-min/week = Days of vigorous-intensity activity × Minutes per day × 8.0 MET + Days of moderate-intensity activity × Minutes per day × 4.0 MET. Grouping criteria: Participants with no moderate or vigorous-intensity activity were assigned to the low-level group; the remaining participants were divided into the moderate and high-level groups based on the median of total MET-min/week in the corresponding domain. TPA: energy expenditure was calculated at a fixed moderate intensity (4 MET), calculation formula: Total MET-min/week = Days of transport-related activity × Minutes per day × 4.0 MET. Grouping criteria: Participants with a total MET-min/week of 0 were assigned to the low-level group; those with a value greater than 0 were divided into the moderate and high-level groups based on the median. The low-level group was used as the reference group for all analyses.

### 2.3 Outcomes

MSDs included nine body sites: neck, shoulders, upper back, elbows, lower back, wrists, hips and buttocks, knees, and ankles and feet. Assessments of all MSDs were conducted using the core screening module of the standardized version of the Nordic Musculoskeletal Questionnaire (NMQ). First released by the Nordic Council of Ministers in 1987, this questionnaire has been validated across multiple occupational populations and is suitable for screening MSDs symptoms in occupational epidemiological studies. The occurrence of symptoms in the nine body sites was investigated through the question “In the past year, have you experienced pain or discomfort in any of the following body parts?”; individuals who reported pain or discomfort in at least one site were defined as MSDs cases. Information on the duration of each pain episode was collected via the question “Please select the duration of each persistent pain episode.” The Numerical Rating Scale (NRS, 0–10 points) recommended by the International Association for the Study of Pain (IASP) was used to quantify pain intensity through the question “Please rate the degree of pain or discomfort in each body part (0 points = no pain, 10 points = worst possible pain).” Meanwhile, the total number of painful sites per individual was counted based on the occurrence of pain in the nine sites, which was categorized into five grades: 0 site, 1 site, 2 sites, 3 sites, and ≥4 sites.

### 2.4 Covariates

Sociodemographic characteristics, lifestyle factors and occupation-related factors were investigated via a questionnaire to control for potential confounding effects. These factors specifically included: age (<25, 25–30, 31–35, >35 years), ethnicity (Han Chinese, ethnic minorities), marital status (unmarried, married, other), educational level (junior high school or below, senior high school or technical secondary school, college or above), body mass index (BMI, underweight, normal weight, overweight, obese), smoking status (non-smoking or occasional smoking, current smoking), alcohol drinking status (non-drinking or occasional drinking, current drinking), working tenure (<5 years, ≥5 years), and monthly income (<5,000 CNY, 5,000–9,999 CNY, >10,000 CNY per month).

### 2.5 Statistical analyses

Categorical variables were presented as frequencies (percentages) [*n* (%)], and continuous variables that conformed to a normal distribution were expressed as mean ± standard deviation. In the main analysis, the MSDs status, pain intensity grade, and pain frequency of each participant were collated. MSDs status was a binary variable, pain intensity grade was a continuous variable, and pain frequency was a polytomous variable. Firstly, the chi-square test was used to compare whether there were statistically significant differences in the distribution of participants’ different characteristics across the three types of physical activity (OPA, LTPA, TPA). Three exposure variables (OPA, LTPA, TPA) were taken as independent variables, and MSDs-related outcomes (pain occurrence, pain intensity, and pain frequency) in the nine body sites were used as dependent variables to construct logistic regression, multiple linear regression, and polytomous regression models. Odds ratios (OR) with 95% confidence intervals (95% CI) and beta coefficients (*β*) with 95% CI were used to quantify the strength of associations between different types of PA and pain occurrence, pain intensity, and pain frequency in the nine body sites. Furthermore, the number of painful sites was categorized into five grades: 0 site, 1 site, 2 sites, 3 sites, and ≥4 sites. Polytomous logistic regression was used to analyze the associations of different types of PA (OPA, LTPA, TPA) with the cumulative number of MSDs-affected sites. All regression models were adjusted for potential confounding factors, including age, ethnicity, marital status, educational level, monthly income, smoking status, drinking status, body mass index (BMI), and length of service. Multiple testing correction was performed using the Benjamini-Hochberg method with a false discovery rate (FDR) of 0.05 to control Type I error. Before constructing the regression models, multicollinearity testing was conducted (variance inflation factor VIF < 10 was considered to indicate no multicollinearity), and all VIF values were <5. All statistical analyses were performed using R software (Version 4.2.1). All statistical analyses were performed using R software (Version 4.2.1).

### 2.6 Ethics approval and consent to participate

This study was approved by the Medical Ethics Committee of Shenzhen Prevention and Treatment Center for Occupational Diseases (Approval No. LL2020-34). All participants provided written informed consent prior to data collection.

## 3 Results

### 3.1 Basic characteristics

The basic characteristics of OPA, LTPA and TPA among 5,522 firefighters are presented in Table 1. In this cross-sectional study, the shoulder had the highest pain prevalence (72.12%, 4,195), followed by the lower back (47.05%, 2,737) and the neck (33.76%, 1,964). The hips and buttocks had the lowest pain prevalence (8.41%, 489), and the pain prevalence of the remaining body sites ranged from 8.41 to 32.03%. For pain intensity, the knees had the highest mean value (1.22 ± 2.25), while the elbows had the lowest (0.26 ± 1.14); the mean pain intensity across all body sites ranged from 0.26 to 1.22 (Supplementary material).

**Table 1:** Baseline characteristics of participants.

Variables	OPA			*p*	LTPA			*p*	TPA			*p*
	Low (*n* = 3,245)	Moderate (*n* = 916)	High (*n* = 1,361)		Low (*n* = 2,765)	Moderate (*n* = 1,466)	High (*n* = 1,291)		Low (*n* = 3,236)	Moderate (*n* = 1,084)	High (*n* = 1,202)	
Number of subjects, *n*												
Age (years), *n* (%)				**<0.001**				**<0.001**				0.089
<25	681 (21.00)	195 (21.30)	334 (24.50)		606 (21.9)	308 (21.0)	296 (22.9)		712 (22.0)	223 (20.6)	275 (22.9)	
25–30	1,345 (41.40)	390 (42.60)	642 (47.20)		1,171 (42.4)	583 (39.8)	623 (48.3)		1,416 (43.8)	449 (41.4)	512 (42.6)	
31–35	627 (19.30)	175 (19.10)	222 (16.30)		528 (19.1)	298 (20.3)	198 (15.3)		610 (18.9)	203 (18.7)	211 (17.6)	
>35	592 (18.20)	156 (17.00)	163 (12.00)		460 (16.6)	277 (18.9)	174 (13.5)		498 (15.4)	209 (19.3)	204 (17.0)	
Ethnicity, *n* (%)				0.203								0.391
Han Chinese	3,120 (96.10)	879 (96.00)	1,293 (95.00)		2,666 (96.4)	1,400 (95.5)	1,226 (95.0)	0.073	3,105 (96.0)	1,031 (95.1)	1,156 (96.2)	
Ethnic minorities	125 (3.90)	37 (4.00)	68 (5.00)		99 (3.6)	66 (4.5)	65 (5.0)		131 (4.0)	53 (4.9)	46 (3.8)	
Marital status, *n* (%)				**0.021**				**0.039**				0.480
Unmarried	1845 (56.90)	527 (57.50)	844 (62.00)		1,610 (58.2)	815 (55.6)	791 (61.3)		1887 (58.3)	609 (56.2)	720 (59.9)	
Married	1,355 (41.80)	375 (40.90)	496 (36.40)		1,118 (40.4)	629 (42.9)	479 (37.1)		1,301 (40.2)	458 (42.3)	467 (38.9)	
Other	45 (1.40)	14 (1.50)	21 (1.50)		37 (1.3)	22 (1.5)	21 (1.6)		48 (1.5)	17 (1.6)	15 (1.2)	
Education, *n* (%)				0.205				**<0.001**				0.684
Lower secondary or below	95 (2.90)	28 (3.10)	31 (2.30)		87 (3.1)	39 (2.7)	28 (2.2)		91 (2.8)	31 (2.9)	32 (2.7)	
Upper secondary/technical	1,374 (42.30)	365 (39.80)	541 (39.80)		1,211 (43.8)	538 (36.7)	531 (41.1)		1,358 (42.0)	446 (41.1)	476 (39.6)	
Tertiary education	1776 (54.70)	523 (57.10)	789 (58.00)		1,467 (53.1)	889 (60.6)	732 (56.7)		1787 (55.2)	607 (56.0)	694 (57.7)	
BMI, *n* (%)				0.421				0.622				**0.033**
Underweight	1,490 (45.90)	408 (44.50)	619 (45.50)		1,248 (45.1)	676 (46.1)	593 (45.9)		1,479 (45.7)	501 (46.2)	537 (44.7)	
Normal weight	1,043 (32.10)	302 (33.00)	458 (33.70)		933 (33.7)	460 (31.4)	410 (31.8)		1,078 (33.3)	343 (31.6)	382 (31.8)	
Overweight	569 (17.50)	177 (19.30)	235 (17.30)		470 (17.0)	274 (18.7)	237 (18.4)		546 (16.9)	212 (19.6)	223 (18.6)	
Obesity	143 (4.40)	29 (3.20)	49 (3.60)		114 (4.1)	56 (3.8)	51 (4.0)		133 (4.1)	28 (2.6)	60 (5.0)	
Smoking status, *n* (%)								**0.032**				0.499
Non-smoking	2077 (64.00)	529 (57.80)	789 (58.00)	<0.001	1715 (62.0)	925 (63.1)	755 (58.5)		1981 (61.2)	658 (60.7)	756 (62.9)	
Smoking	1,168 (36.00)	387 (42.20)	572 (42.00)		1,050 (38.0)	541 (36.9)	536 (41.5)		1,255 (38.8)	426 (39.3)	446 (37.1)	
Alcohol drinking status, *n* (%)								0.589				0.269
Non-drinking	3,230 (99.50)	904 (98.70)	1,352 (99.30)	0.019	2,749 (99.4)	1,457 (99.4)	1,280 (99.1)		3,215 (99.4)	1,080 (99.6)	1,191 (99.1)	
Drinking	15 (0.50)	12 (1.30)	9 (0.70)		16 (0.6)	9 (0.6)	11 (0.9)		21 (0.6)	4 (0.4)	11 (0.9)	
Working years, *n* (%)				0.439				0.332				0.069
<5	2,528 (77.90)	706 (77.10)	1,037 (76.20)		2,132 (77.1)	1,153 (78.6)	986 (76.4)		2,473 (76.4)	865 (79.8)	933 (77.6)	
≥5	717 (22.10)	210 (22.90)	324 (23.80)		633 (22.9)	313 (21.4)	305 (23.6)		763 (23.6)	219 (20.2)	269 (22.4)	
Monthly income								**<0.001**				0.115
<5,000 yuan	557 (17.2)	165 (18.0)	225 (16.5)	<0.001	478 (17.3)	232 (15.8)	237 (18.4)		539 (16.7)	199 (18.4)	209 (17.4)	
5,000 ~ 9,999 yuan	2,406 (74.1)	644 (70.3)	887 (65.2)		2019 (73.0)	2019 (73.0)	859 (66.5)		2,324 (71.8)	778 (71.8)	835 (69.5)	
>10,000 yuan	282 (8.7)	107 (11.7)	249 (18.3)		268 (9.7)	175 (11.9)	195 (15.1)		373 (11.5)	107 (9.9)	158 (13.1)	

Bold values indicate *p* < 0.05, representing statistical significance.

There were statistically significant differences in the distribution of OPA across age, marital status, smoking status, alcohol drinking status and monthly income (*p* < 0.05). Individuals with high OPA were more likely to be younger, unmarried, current smokers, and have a higher monthly income. For LTPA, statistically significant distribution differences were observed across age, marital status, educational level, smoking status and monthly income (*p* < 0.05); individuals with high LTPA were more likely to be younger, unmarried, have a higher educational level, be current smokers, and have a higher monthly income. A statistically significant difference was found in the distribution of TPA across BMI categories (*p* < 0.05), and individuals with high TPA had the lowest proportion of those with low BMI.

### 3.2 Associations of different types of PA with the onset and pain intensity of MSDs

**Figure 1:**
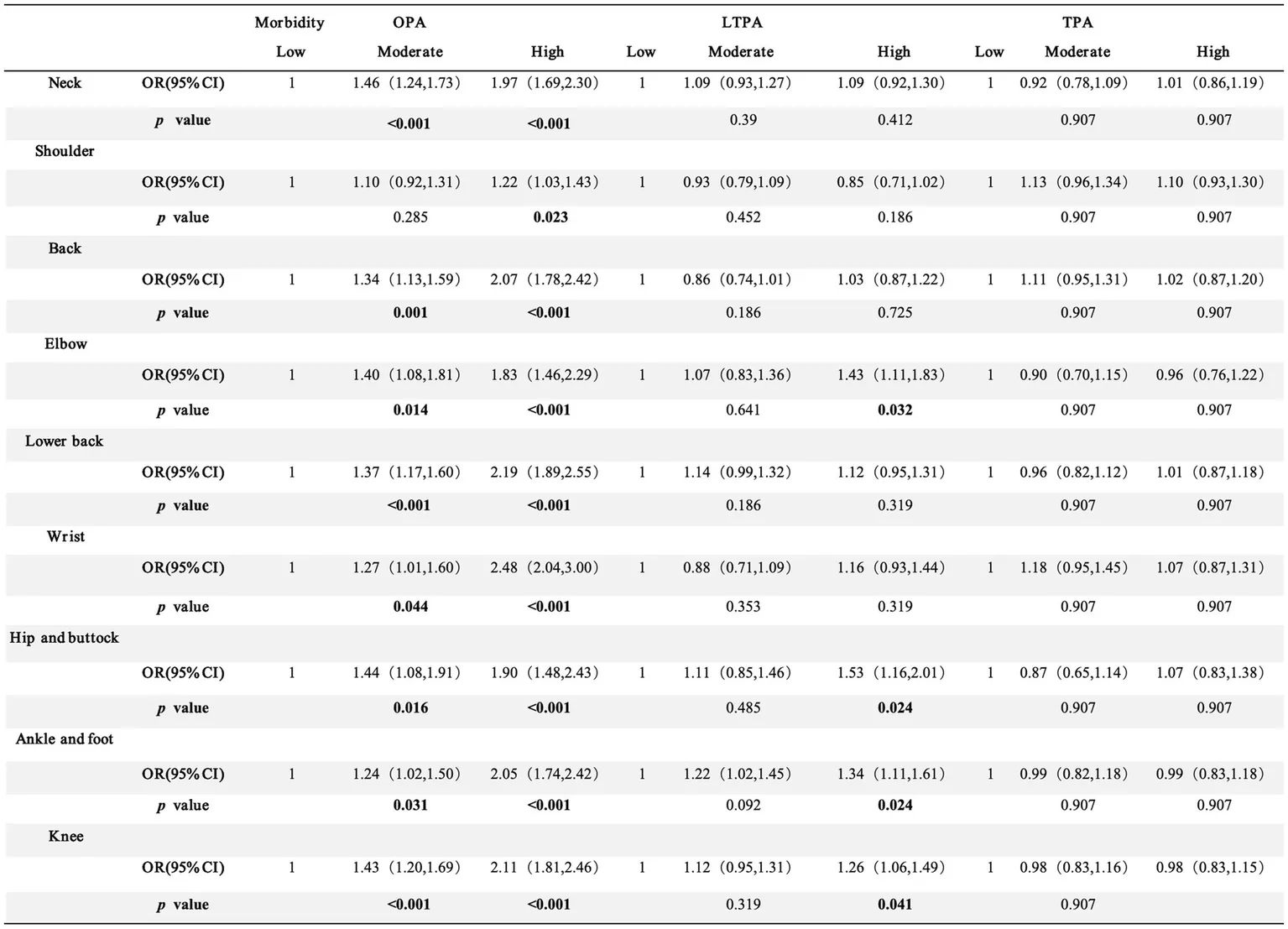
Associations of OPA, LTPA, and TPA with MSDs occurrence.

**Figure 2:**
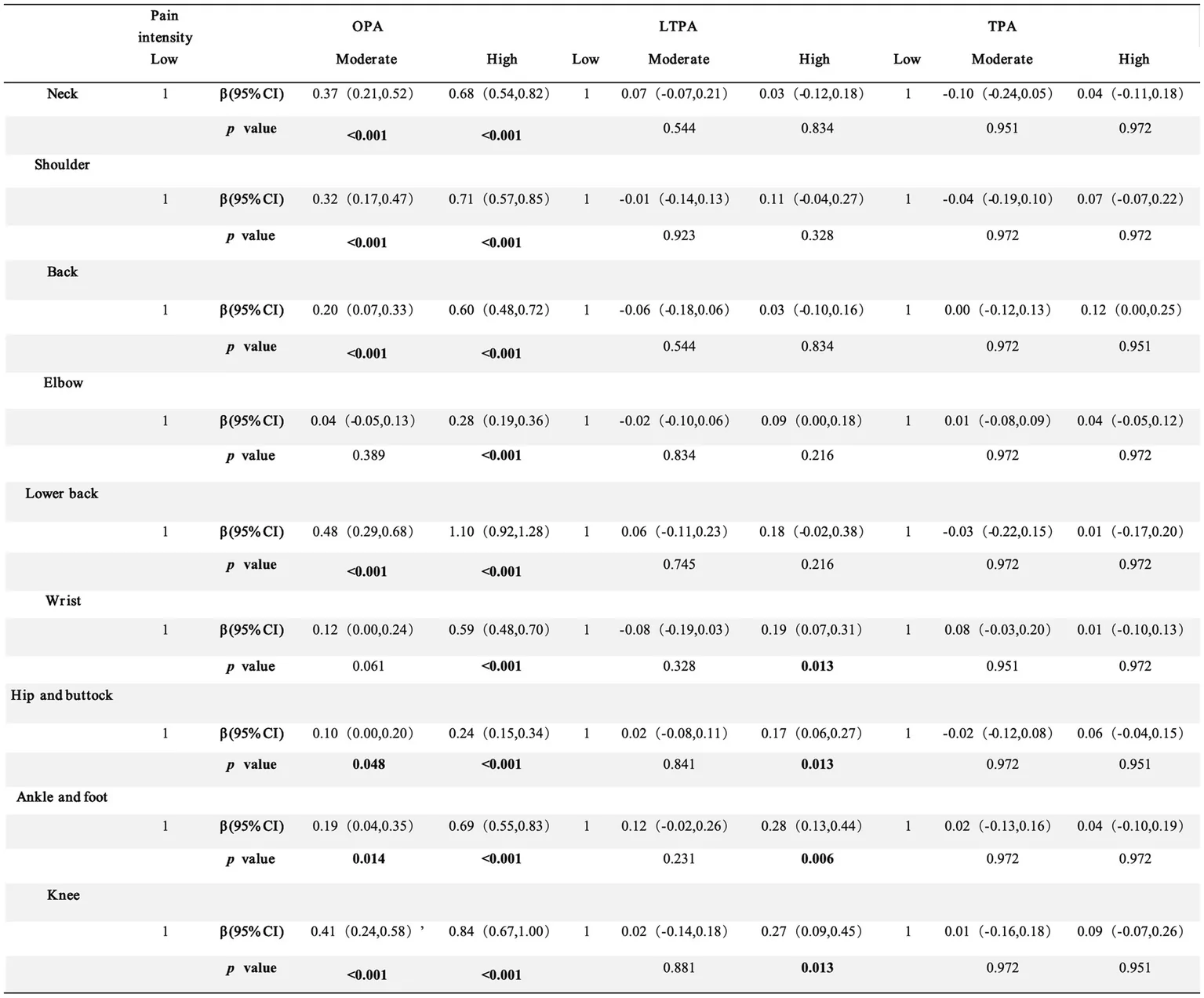
Associations of OPA, LTPA, and TPA with MSDs pain intensity.

With the increase in OPA intensity, the risk of MSDs onset at all nine body sites increased significantly (OR = 1.22 ~ 2.48, *p* < 0.05), and the pain intensity at these nine sites also rose markedly (*β* = 0.24 ~ 1.10, *p* < 0.001). Among these, lower back pain intensity was the most affected (*β* = 1.10, *p* < 0.001). Compared with individuals with low-level OPA, those with high-level OPA had a 1.10-point increase in lower back pain intensity, followed by the knees (*β* = 0.84, *p* < 0.001). Only high-level LTPA was associated with an elevated risk of MSDs onset in the elbows, hips and buttocks, ankles and feet, and knees (OR = 1.26 ~ 1.53, *p* < 0.05), and it significantly increased the pain intensity in the wrists, hips and buttocks, ankles and feet, and knees (*β* = 0.17 ~ 0.28, *p* < 0.05), with the ankles and feet being the most prominent (*β* = 0.28, *p* = 0.006). In contrast, TPA showed no statistical association with MSDs onset or pain intensity at any body site (*p* > 0.05) (Figures 1, 2).

### 3.3 Associations of different types of PA with the pain frequency of MSDs

**Figure 3:**
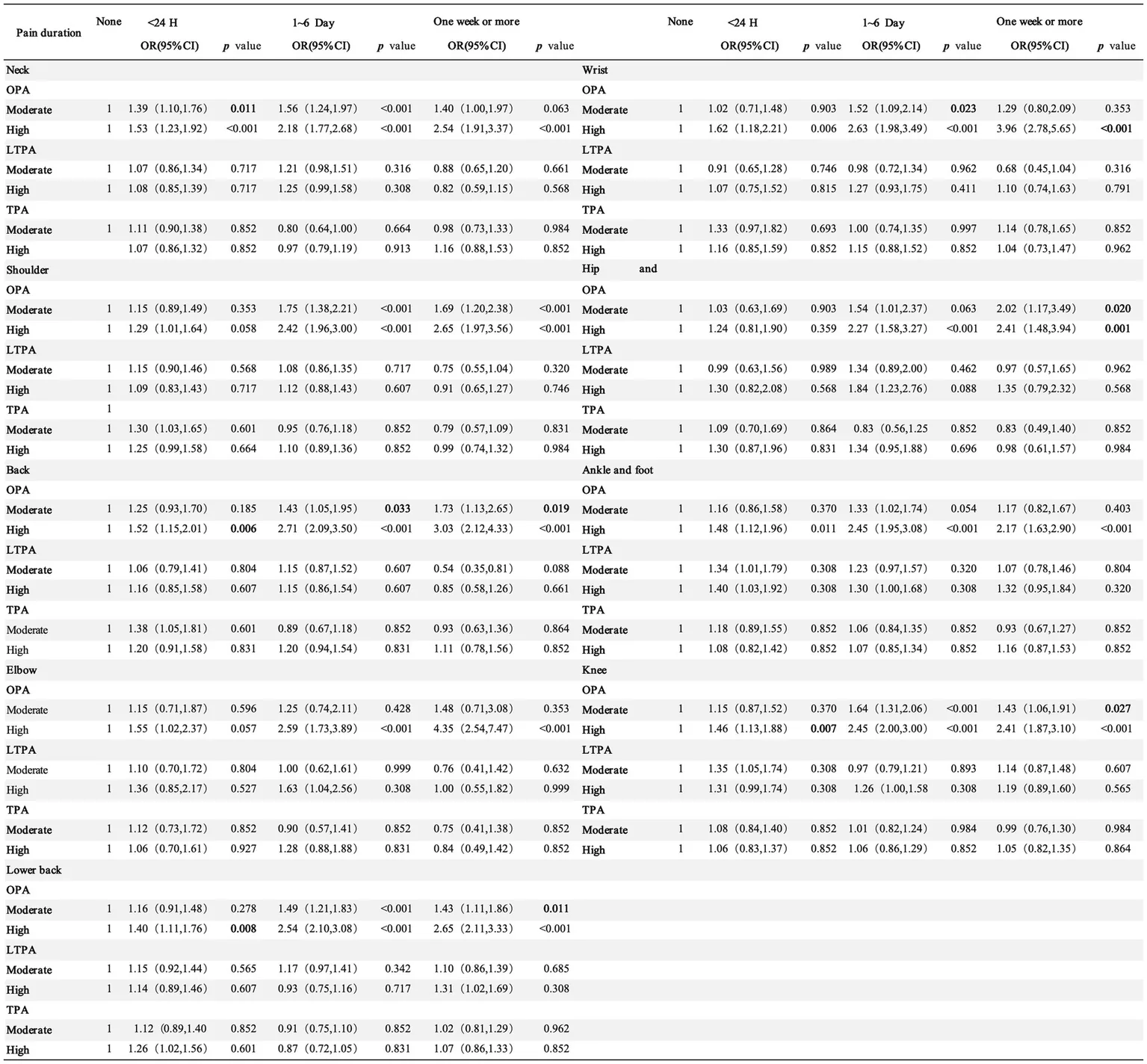
Associations of OPA, LTPA, and TPA with MSDs pain frequency.

OPA was associated with an elevated risk of prolonged pain duration at all nine body sites. The elevated risk was the most prominent for pain lasting ≥1 week, followed by pain lasting 1–6 days. OPA exerted the most significant effects on the risk of prolonged pain frequency at four sites: the upper back (OR = 3.30, *p* < 0.001 for pain lasting ≥1 week), the elbows (OR = 4.35, *p* < 0.001 for pain lasting ≥1 week), the lower back (OR = 2.65, *p* < 0.001 for pain lasting ≥1 week), and the wrists (OR = 3.96, *p* < 0.001 for pain lasting ≥1 week). No significant associations were observed for LTPA or TPA with prolonged pain frequency at any site (Figure 3).

### 3.4 Associations of different types of PA with the cumulative number of MSDs-affected painful sites

We further investigated the association between PA and the cumulative number of MSD-affected painful sites (Table 2). OPA was the core risk factor for an increased cumulative number of MSDs-affected painful sites, with the risk becoming more prominent as the number of painful sites rose. In the high-level OPA group, the risk of pain at 2 sites increased by 69% (OR = 1.69, 95% CI: 1.20 ~ 2.38, *p* = 0.003); the risk of pain at 3 sites increased by 135% (OR = 2.35, 95% CI: 1.65 ~ 3.34, *p* < 0.001); the risk of pain at ≥4 sites increased by 290% (OR = 3.90, 95% CI: 2.74 ~ 5.54, *p* < 0.001). In the moderate-level OPA group, the risk of pain at 2 sites increased by 80% (OR = 1.80, 95% CI: 1.28 ~ 2.53, *p* = 0.001); the risk of pain at 3 sites increased by 93% (OR = 1.93, 95% CI: 1.35 ~ 2.75, *p* < 0.001); the risk of pain at ≥4 sites increased by 133% (OR = 2.33, 95% CI: 1.63 ~ 3.33, *p* < 0.001). No statistically significant associations were found between LTPA, TPA and the risk of pain at multiple sites.

**Table 2:** Associations of OPA, LTPA, and TPA with MSDs at nine body sites.

Cumulative painful sites	*n* (%)	OR (95% CI) and *p* value	OPA			LTPA			TPA		
			Low	Moderate	High	Low	Moderate	High	Low	Moderate	High
0	540 (9.78)		1			1			1		
1	1,339 (24.25)	OR (95% CI)	1	1.25 (0.89, 1.76)	1.13 (0.80, 1.58)	1	1.21 (0.87, 1.69)	0.92 (0.65, 1.28)	1	1.0 9(0.78, 1.53)	1.05 (0.75, 1.48)
		*p* value	1	0.198	0.502	1	0.265	0.589	1	0.701	0.782
2	1,155 (20.92)	OR (95% CI)	1	1.80 (1.28, 2.53)	1.69 (1.20, 2.38)	1	1.22 (0.87, 1.71)	0.79 (0.56, 1.12)	1	0.99 (0.70, 1.40)	1.02 (0.72, 1.43)
		*p* value	1	**0.001**	**0.003**	1	0.251	0.187	1	0.958	0.915
3	903 (16.35)	OR (95% CI)	1	1.93 (1.35, 2.75)	2.35 (1.65, 3.34)	1	1.39 (0.99, 1.95)	0.91 (0.65, 1.27)	1	1.17 (0.82, 1.65)	1.13 (0.80, 1.59)
		*p* value	1	**<0.001**	**<0.001**	1	0.054	0.572	1	0.391	0.505
≥4	1,585 (28.70)	OR (95% CI)	1	2.33 (1.63, 3.33)	3.90 (2.74, 5.54)	1	1.26 (0.89,1.78)	1.13 (0.80,1.58)	1	1.05 (0.74,1.49)	1.00 (0.70,1.42)
		*p* value	1	**<0.001**	**<0.001**	1	0.191	0.501	1	0.791	0.989

Bold values indicate *p* < 0.05, representing statistical significance.

## 4 Discussion

The results of this study indicated that the association between OPA and MSDs was the most prominent: OPA showed a clear dose–response relationship with the occurrence and pain intensity of MSDs, and high OPA was also significantly associated with an increased risk of pain frequency in MSDs involving all nine body sites. High LTPA was only related to the occurrence and pain intensity of MSDs in specific body sites, while no independent association between TPA and MSDs was observed. In addition, a significant correlation was found between the cumulative number of MSDs-affected sites and high OPA. These findings collectively highlight the complex relationships among OPA, LTPA, and TPA. Among the three types of PA exposure, high OPA was an independent risk factor for MSDs onset, which is consistent with the conclusions of most previous studies—high-workload OPA contribute to lower back, neck-shoulder, upper limb and sciatic nerve pain (30–32). We extended this evidence base by demonstrating that high OPA may also significantly exacerbate pain intensity and prolong pain frequency. Long-term occupational activities are associated with an increased risk of cumulative musculoskeletal injuries, which in turn are related to the severity of the condition. As frontline rescuers, firefighters are prone to such cumulative injuries and chronic pain induced by high OPA. These issues not only directly elevate the risk of MSDs but also impair the accuracy and response speed of firefighters’ work; indirectly, they increase the probability of occupational injuries caused by physical functional limitations, thus becoming a critical concern in occupational health and safety. High LTPA exhibited distinct site-specificity: it was an independent risk factor for the onset of MSDs in the elbows, hips and buttocks, ankles and feet, and knees, and particularly exacerbated pain intensity in the ankles and feet as well as the knees. This may be primarily attributed to the distinct physiological functions and load-bearing characteristics of the ankles/feet and knees. As a typical weight-bearing joint, the knee mainly bears vertical impact loads during movement. Prolonged high-intensity activity directly aggravates articular cartilage wear due to such impact loads. In contrast, the ankles and feet are primarily responsible for shock absorption and stability, bearing repetitive tensile loads rather than acute impact (33). Moderate-intensity LTPA in this study (e.g., prolonged brisk walking and cycling) mostly consisted of sustained repetitive movements, which are more likely to induce chronic fatigue-related injuries in the ankles and feet. Second, high-intensity LTPA may be associated with an increased risk of local inflammation. Excessive exercise can upregulate the expression of inflammatory factors such as IL-1β and COX2, reduce the pain threshold, and simultaneously impair the stabilizing effect of muscles surrounding the joints, thereby being associated with enhanced pain perception in musculoskeletal tissues throughout the body (34). This finding is supported by the study of Riihimäki et al. (35). Notably, the observed association between high LTPA and MSDs risk in this study does not negate the potential protective effects of LTPA reported in previous research. In most prior studies, LTPA exposure was either low-intensity or a mixed dose of “low-intensity plus appropriate moderate-to-vigorous intensity,” with no distinction made between the independent effects of low-intensity and moderate-to-vigorous intensity LTPA (32, 36). We clarified the risk effects of pure high-intensity LTPA alone, and our conclusions are also supported by similar previous studies: a cross-sectional study of office workers by Putsa et al. (33) found that ≥150 min of weekly moderate-to-vigorous LTPA was associated with an increased risk of MSDs onset. Additionally, studies by Riihimäki et al. (35) on machine operators and Jakobsen et al. on healthcare workers have also indicated the potential harms of high-level LTPA on MSDs, even among general populations with different occupational backgrounds (37). These results suggest that interventions targeting leisure-time physical activity among occupational populations with high physical workload should follow the dose–response principle. The general recommendation of “encouraging more exercise during leisure time” may not be appropriate for firefighter populations engaged in rescue work. Therefore, exercise modes should be appropriately optimized to avoid secondary loading of the musculoskeletal system caused by non-occupational high-intensity physical activity.

Unlike OPA and LTPA, no independent association between TPA and MSDs was identified in our study. TPA in most populations is dominated by short-duration walking and cycling, with an intensity lower than that of both LTPA and OPA, which may be insufficient to reach the threshold of sustained repetitive loading or high-intensity impact loading required to trigger MSDs (32, 37, 38). Meanwhile, TPA includes diverse forms such as walking and cycling, with substantial differences in load characteristics across these forms (28); such heterogeneity may weaken or offset unidirectional association signals. The results of our study highlight the need to develop targeted intervention strategies and further clarify the distinct modes of action of the three types of PA in the pathogenesis of MSDs. In addition, our analysis revealed that OPA was a key risk factor for an increased cumulative number of painful sites, and the association was stronger with a greater number of painful sites. This may be explained by the fact that the sustained physical workload associated with high OPA is linked to muscle fatigue, joint wear, and metabolic stress, and such injury exhibits a cumulative effect. Such damage cannot be fully restored by short-term rest, ultimately leading to an elevated risk of multisite pain (31, 32, 39). This refined, stratified association pattern has not been clearly reported previously, and we further elucidated the cumulative damage induced by high OPA. Multisite MSDs comorbidity is not merely a simple superposition of pain in individual body sites. The comprehensive functional deficits resulting from such comorbidity often exert an amplifying effect, which substantially increases the occupational disease burden among firefighters, reduces occupational quality of life, elevates absenteeism and medical costs, and impairs the overall operational effectiveness of the rescue team. It also represents one of the major contributors to early service exit among firefighters, imposing a long-term hidden burden on this occupational group.

Collectively, the present study indicates that the prevention and control of MSDs among firefighters require tailored strategies according to different types of physical activity. Future studies should further investigate the threshold inflection points of risk for various PA exposures and evaluate the effectiveness of intervention strategies targeting different physical activity profiles.

## 5 Strengths and limitations

To the best of our knowledge, the present study is the first large-sample investigation (5,522 cases; existing studies on firefighters’ MSDs were mostly small with 70–1,311 cases) to explore the independent associations of domain-specific PA with MSDs onset, pain intensity, and pain frequency simultaneously. This study focused on firefighters as a highly physically demanding occupational group, covering nine affected body sites and conducting cumulative pain assessment. Some previous studies only treated PA as a global exposure and analyzed its association with a single MSDs outcome (either onset or pain intensity), without simultaneously integrating onset, pain intensity, and pain frequency for comprehensive analysis. The core innovation of this study is that we overcame the limitation of treating PA as a global exposure by strictly distinguishing different PA domains, thereby filling the theoretical gap regarding the differential effects of domain-specific PA on MSDs in previous research. We further clarified the independent risk effects of moderate-to-high intensity LTPA on MSDs in specific body sites, providing evidence for the health risks of high-level LTPA. Meanwhile, we are the first to reveal the cumulative risk effect of high OPA on MSDs pain, offering a novel scientific basis for understanding multisite pain risk in this population.

This study also has several limitations. First, as a cross-sectional study, it only reveals correlations between variables, cannot determine causal relationships, and is unable to rule out reverse causality. Second, although exposure variables and outcome indicators were obtained via standard scales, they remain subjective, which may lead to recall bias or reporting bias and thus make it difficult to accurately capture the actual exposure levels and symptom severity in full. Additionally, we must acknowledge that in our main analyses, we did not further refine the temporal distribution characteristics of different types of PA (e.g., whether LTPA was concentrated exercise in the form of “weekend warrior” behavior) or differences in exercise modalities. Nor did we include potential mediating or moderating variables such as muscle strength, cardiopulmonary endurance and inflammatory cytokines, which limits the in-depth interpretation of the underlying association mechanisms.

## 6 Conclusion

This study found the OPA and LTPA were significantly associated with MSDs at nine body sites among high-workload firefighters. Specifically, high-intensity OPA was the core and strongest risk factor for the onset of MSDs, as well as pain intensity, pain frequency and the cumulative number of painful sites in firefighters; high-intensity LTPA was only associated with the onset and pain intensity of MSDs at specific body sites, while TPA had no significant independent association with MSDs. This finding provides empirical evidence for the specific effects of OPA and LTPA on MSDs in high-workload occupational populations, demonstrating distinct domain-specific differences in the associations between different types of physical activity and MSDs, and also clarifies the potential risks of high-intensity LTPA for MSDs at specific body sites. Therefore, comprehensive and targeted policies and intervention measures should be implemented to scientifically regulate the exposure levels of physical activity in high-workload occupational populations, thereby preventing the onset and exacerbation of MSDs symptoms.

## Data Availability

The datasets presented in this article are not readily available because data are unavailable due to privacy or ethical restrictions.
