# Correction: Associations of different types of physical activity (PA) with musculoskeletal disorders (MSDs) at nine body sites in firefighters

**DOI:** 10.3389/fpubh.2026.1976271

**Published:** 2026-09-29

**Authors:** Wenli Gan, Shaofan Weng, Wei Zhou, Naixing Zhang, Huan Guo, Dafeng Lin

**Affiliations:** 1 Department of Epidemiology and Biostatistics, School of Public Health, Jilin University, Changchun, Jilin, China; 2 Laboratory of Occupational Health Epidemiology, Shenzhen Prevention and Treatment Center for Occupational Diseases, Shenzhen, Guangdong, China

**Keywords:** firefighters, musculoskeletal disorders (MSDs), occupational health, occupational physical activity, physical activity (PA)

Authors Shaofan Weng, Wei Zhou, Naixing Zhang, Huan Guo, Dafeng Lin were erroneously assigned as equal contributing authors.

The original version of this article has been updated.

