# Supplementary material for: Associations of different types of physical activity (PA) with musculoskeletal disorders (MSDs) at nine body sites in firefighters
Source: Front Public Health. 2026 Mar 30;14:1804818. doi: 10.3389/fpubh.2026.1804818 (PMC13071070; doi:10.3389/fpubh.2026.1804818)
Supplement: Supplementary file 1 [file Supplementary_file_1.docx]

| **Supplement 1** | | |
| --- | --- | --- |
| **Physical Activity** | | |
|  |  |  |
| **OPA** | | |
| Do your work involve vigorous-intensity activities that cause a substantial increase in breathing or heart rate (e.g., lifting/carrying heavy objects, digging, construction work) and last for at least 10 minutes continuously? (Show card) | Yes 1  No 2 | Calculation basis: Calculation formula from WHO Global Physical Activity Guidelines  Standardization of total energy expenditure of occupational activities with different intensities and durations: Total MET-min/week of occupational activities (energy expenditure) = Days of vigorous activity × Minutes per day × 8.0 + Days of moderate activity × Minutes per day × 4.0  Grouping criteria: Participants with no moderate or vigorous activity were classified as low; the rest were classified as moderate or high based on the median of total MET-min/week of occupational activities |
| How many days per typical work week do you engage in vigorous-intensity physical activity as part of your work? | Number of days |  |
| How much time do you usually spend on vigorous-intensity activities per work day? | Hours |  |
| Do your work involve moderate-intensity activities that cause a slight increase in breathing or heart rate (e.g., brisk walking for at least 10 minutes continuously, or lifting/carrying light loads)? (Show card) | Yes 1  No 2 |  |
| How many days per typical week do you engage in moderate-intensity physical activity as part of your work? | Number of days |  |
| How much time do you usually spend on moderate-intensity activities per work day? | Hours |  |
| **TPA** | | |
| Now I would like to ask how you usually travel to and from places such as work, shopping malls, markets, and places of worship? | | |
| Do you walk or cycle (bicycle) for at least 10 minutes continuously for round trips to destinations? | Yes 1  No 2 | Calculation basis: Total MET-min/week of transport-related activities (energy expenditure) = Number of days × Minutes per day × 4.0 (fixed as moderate intensity)  Grouping criteria: Participants with a value of 0 were classified as low; the rest were classified as moderate or high based on the median of total MET-min/week of transport-related activities. |
| How many days per typical week do you walk or cycle for at least 10 minutes continuously for round trips to destinations per day? | Number of days |  |
| How much time do you usually spend walking or cycling for travel per day? | Hours |  |
| **LTPA** |  |  |
| Do you engage in vigorous-intensity sports, fitness or leisure activities (e.g., running, football) that cause a marked increase in breathing or heart rate and last for at least 10 minutes continuously? (Show card) | Yes 1  No 2 | Calculation basis: Standardization of total energy expenditure of leisure-time activities with different intensities and durations: Total MET-min/week of leisure-time activities (energy expenditure) = Days of vigorous activity × Minutes per day × 8.0 + Days of moderate activity × Minutes per day × 4.0  Grouping criteria: Participants with no moderate or vigorous activity were classified as low; the rest were classified as moderate or high based on the median of total MET-min/week of leisure-time activities. |
| How many days per typical week do you engage in vigorous-intensity sports, fitness or leisure activities?  Number of days | Number of days |  |
| How much time do you usually spend on vigorous-intensity sports, fitness or leisure activities per typical day? | Hours |  |
| Do you engage in moderate-intensity sports, fitness or leisure activities (e.g., brisk walking, cycling, swimming, volleyball) that cause a slight increase in breathing or heart rate and last for at least 10 minutes continuously? (Show card) | Yes 1  No 2 |  |
| How many days per typical week do you engage in moderate-intensity sports, fitness or leisure activities?  Number of days | Number of days |  |
| How much time do you usually spend on moderate-intensity sports, fitness or leisure activities per typical day? | Hours |  |

**Supplement 2**

| Body site | Painful cases (n) | Pain rate (%) | Pain grade (Mean ± SD, %) |
| --- | --- | --- | --- |
| Neck | 1964 | 33.76 | 1.16 ± 2.02 |
| Shoulder | 4195 | 72.12 | 1.08 ± 2.04 |
| Back | 1863 | 32.03 | 0.68 ± 1.71 |
| Elbow | 609 | 10.47 | 0.26 ± 1.14 |
| Lower back | 2737 | 47.05 | 0.56 ± 1.59 |
| Wrist | 872 | 14.99 | 0.56 ± 1.59 |
| Hip and buttock | 489 | 8.41 | 0.34 ± 1.31 |
| Ankle and foot | 1286 | 22.11 | 0.87 ± 1.96 |
| Knee | 1775 | 30.51 | 1.22 ± 2.25 |
